# MIIP remodels Rac1-mediated cytoskeleton structure in suppression of endometrial cancer metastasis

**DOI:** 10.1186/s13045-016-0342-6

**Published:** 2016-10-19

**Authors:** Yingmei Wang, Limei Hu, Ping Ji, Fei Teng, Wenyan Tian, Yuexin Liu, David Cogdell, Jinsong Liu, Anil K. Sood, Russell Broaddus, Fengxia Xue, Wei Zhang

**Affiliations:** 1 Department of Gynecology and Obstetrics, Tianjin Medical University General Hospital, Tianjin, China; 2 Department of Pathology, The University of Texas MD Anderson Cancer Center, Houston, TX USA; 3 Department of Systems Biology, The University of Texas MD Anderson Cancer Center, Houston, TX USA; 4 Department of Bioinformatics, The University of Texas MD Anderson Cancer Center, Houston, TX USA; 5 Department of Gynecologic Oncology and Reproductive Medicine, The University of Texas MD Anderson Cancer Center, Houston, TX USA; 6 Center for RNAi and Non-Coding RNA, The University of Texas MD Anderson Cancer Center, Houston, TX USA; 7 Department of Cancer Biology, Comprehensive Cancer Center of Wake Forest Baptist Medical Center, Winston-Salem, NC 27157 USA; 8 Present Address: Department of Biochemistry and Molecular Biology, The University of Texas Medical Branch, Galveston, TX USA

**Keywords:** Endometrial cancer, MIIP, Migration, Rac1/PAK1 pathway, Tumor suppressor gene

## Abstract

**Background:**

Endometrial carcinoma (EC) is one of the most common malignancies of the female reproductive system. Migration and invasion inhibitory protein (MIIP) gene was recently discovered candidate tumor suppress gene which located at chromosome 1p36.22. 1p36 deletion was found in many types of tumor including EC. In the present study, we will determine the role and mechanism of MIIP in EC metastasis.

**Methods:**

Immunohistochemistry was used to measure MIIP expression in normal and EC tissue. Both gain-of-function (infection) and loss-of-function (siRNA) assays were used to alter MIIP expression levels. The effect of MIIP on cell migration and invasion was measured by transwell assay. F-actin immunofluorescence staining was used to observe the cell morphology. The activation of GTP-loaded Rac1 was evaluated by Rac activity assay kit. Immunoprecipitation/WB was used to measure the interaction between MIIP and PAK1.

**Results:**

We demonstrate that MIIP expression was significantly decreased in EC patients comparing to the normal ones, and decreased MIIP expression in EC tissues is associated with deep myometrial invasion, advanced stage, and the presence of lymph node metastasis. Using both gain-of-function (infection) and loss-of-function (siRNA) assays, we show that MIIP markedly blocked EC cell migration, whereas loss of MIIP led to increase in EC cell migration. We demonstrate that elevated expression of *MIIP* resulted in cytoskeleton reorganization with decreased formation of lamellipodia. We also provide evidence that MIIP is a key molecule in directing Rac1 signaling cascades in EC. Ectopically expressed MIIP consistently competed with Rac1-GTP for binding with the PAK1 p21-binding domain. Our data show that MIIP and PAK1 bind each other and that a C-terminal polyproline domain of MIIP is required for PAK1 binding. Deletion of the PAK1-binding domain of MIIP reduced cell migration-inhibiting activity.

**Conclusions:**

MIIP may function as a tumor suppressor gene for endometrial carcinoma. MIIP attenuates Rac1 signaling through a protein interaction network, and loss of this regulator may contribute to EC metastasis.

**Electronic supplementary material:**

The online version of this article (doi:10.1186/s13045-016-0342-6) contains supplementary material, which is available to authorized users.

## Background

Endometrial cancer (EC) is the second most common gynecologic cancer worldwide [1]. The American Cancer Society reports that the number of newly diagnosed EC cases increased from 35,000 to 60,050 in the USA alone between 1987 and 2016; the number of deaths rose from 2900 to 10,470, a 261 % increase [2]. The 5-year survival rate for early stage EC is close to 70 %, but the median survival rate drops to 1 year for patients with advanced stage. Even among patients with apparent early-stage disease, some will go on to develop localized recurrent and distant metastases [3]. However, there has been very little gain in therapeutic efficacy during the past 30 years [4, 5].

Understanding the molecular mechanisms underlying EC migration is among the most important goals of EC research. The molecular mechanisms that account for local or distant metastasis are not well understood. The migration and invasion inhibitory protein, which is encoded by the recently discovered *MIIP* (also termed *IIp45*) gene, located on chromosome 1p36.22 and spanning 12.6 kb of genomic DNA, inhibits migration and invasion of human glioma cells [6]. The *MIIP*-containing chromosome 1p36 region has been shown to be deleted in a wide spectrum of human cancers, including EC [7–9], which suggests that MIIP may also be a negative regulator of EC progression. Previous studies have shown that MIIP inhibits glioma cell migration and invasion through two mechanisms: (1) attenuating insulin-like growth factor-binding protein 2 (IGFBP2)-mediated cell migration [10] and (2) blocking HDAC6 activity and increasing acetylated α-tubulin, which stabilizes microtubules [11]. Like microtubules, the actin cytoskeleton is a major mechanism for cell migration. In migrating cells, growing microtubules that reach into the leading edge promote Rac activation and the formation of short, branched F-actin for lamellipodia formation. cDNA microarray gene expression profiling identified a group of motility-associated genes whose expression was downregulated in MIIP-expressing cells, including Rho GTPase family members, the transcription factor *NF*-*κB* and its downstream target genes [10]. Rac1 is a member of the Rho family of GTPases that induces formation of lamellipodia protrusions and membrane ruffles through interaction with its specific effector, p21-activating kinase (PAK) [12]. The activation of Rac1 and its downstream effectors has been associated with tumor cell migration, invasion, and/or metastasis in breast, ovarian, lung, colorectal, bladder, and ECs [13–19]. However, it is not clear how MIIP modulates the Rho GTPase family members.

In the study presented here, we demonstrate that decreased expression of MIIP was significantly associated with deep myometrial invasion, advanced stage, and the presence of lymph node metastasis in EC. We also show that knockdown of *MIIP* increased EC cell migration, while restoration of *MIIP* inhibited EC cell migration. MIIP expression had a marked impact on lamellipodia formation. Furthermore, we demonstrate that MIIP inhibited EC cell migration through blocking of the Rac1 signal transduction pathway by directly binding to its downstream effector PAK1, and a C-terminal polyproline domain of MIIP is required for PAK1 binding.

## Results

### Decreased MIIP expression is associated with tumorigenesis and progression of EC

**Fig. 1:**
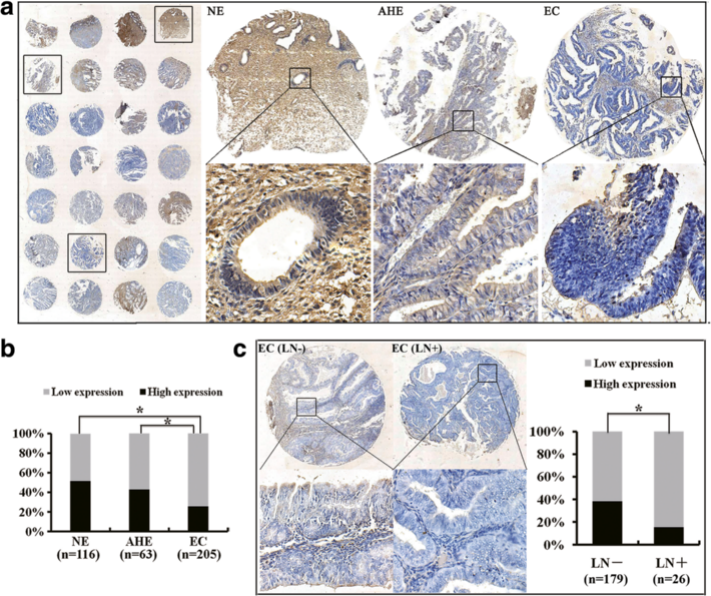
Expression of *MIIP* is reduced in human EC specimens. **a** MIIP expression was evaluated by immunohistochemical staining on TMAs. The respective images in the same TMAs showed that MIIP expression was lower in EC than those in NE and AHE. **b** Statistical analysis revealed that MIIP expression was highest in NE, lower in AHE, and lowest in EC. **c** Loss expression of MIIP was related to lymph node metastasis in EC. *Left panel*: Shown are representative images of MIIP expression in EC tissues with or without lymph node metastasis. *Right panel*: Statistical analysis revealed that low MIIP expression was correlated with lymph node metastasis in EC patients. *Asterisk* indicates *P* < 0.05. See also Table 1

To investigate MIIP’s role in EC tumorigenesis and progression, we first evaluated MIIP protein expression in normal endometrium (NE), atypical hyperplasia endometrium (AHE), and EC using an immunohistochemical analysis on tissue microarrays (TMAs). Among the 384 cases available for the analysis on TMA, MIIP expression was highest in NE (51.72 %, 116 cases), lower in AHE (42.85 %, 63 cases), and lowest in EC (25.85 %, 205 cases, Fig. 1a, b). Detailed clinical correlation analyses revealed that among the 205 EC patients, a low level of MIIP expression was associated with deep myometrial invasion, lymph node metastasis, and advanced FIGO stage (Fig. 1c and Table 1).

**Table 1:** Correlation between MIIP protein expression and pathological parameters of EC

Pathological characteristic	No.	MIIP expression, *n* (%)		*χ* ^2^	*P*
		Low	High		
Histologic subtype					
Endometrioid	183	126 (68.85)	57 (31.15)	1.827	0.176
Non-endometrioid	22	12 (54.55)	10 (45.45)		
Histopathological grade					
1	82	58 (70.73)	24 (29.27)	0.204	0.903
2	77	55 (71.43)	22 (28.57)		
3	24	16 (66.67)	8 (22.33)		
FIGO stage					
I and II	166	109 (65.77)	57 (34.33)	6.930	0.008
III and IV	39	34 (87.28)	5 (12.82)		
Myometrial invasion					
<1/2	141	90 (63.83)	51 (36.17)	7.519	0.006
≥1/2	64	53 (82.01)	11 (17.19)		
Lymph node status					
Negative	179	110 (61.45)	69 (38.55)	5.312	0.021
Positive	26	22 (84.62)	4 (15.38)		

### MIIP inhibits EC cell migration and invasion

**Fig. 2:**
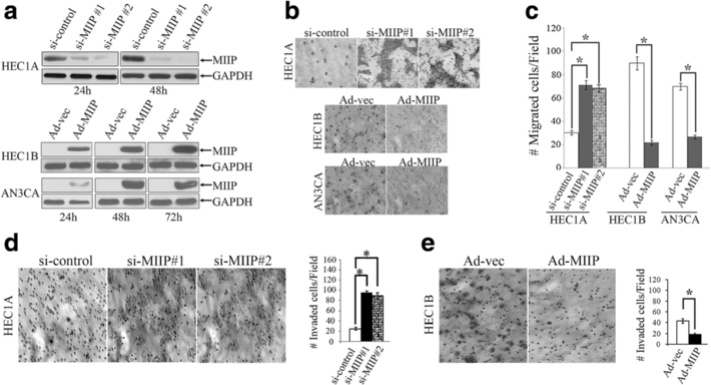
MIIP inhibits EC cell migration and invasion. **a** Western blot shows that MIIP was knocked down by two different siRNAs against *MIIP* when compared to control at 24 and 48 h. And MIIP expression was forced in HEC1B and AN3CA cells by infection with an adenovirus containing *MIIP* (Ad-*MIIP*) or control adenovirus (Ad-Ev) at 24, 48, or 72 h. **b**, **c** Modulation of EC cell migration by MIIP in a transwell migration chamber. **b** Representative photographs revealed knockdown of *MIIP* enhanced HEC1A cell migration and overexpression of *MIIP* inhibited HEC1B and AN3CA cell migration (magnification ×200). **c** Data are expressed as means ± SD of cells per 10 high-power fields from three separate experiments. **d**, **e** Modulation of EC cell invasion by MIIP in a transwell invasion chamber. **d** *Left*: Representative images of cells on the filter surface of HEC1A (×200 magnification). *Right*: Quantitative measurement of invaded HEC1A cells. Data are represented by the mean ± SD of cells per 10 high-power fields from three separate experiments. **e** *Left*: Representative images of cells on the filter surface of HEC1B (×200 magnification). *Right*: Quantitative measurement of invaded HEC1B cells. *Asterisk* indicates *P* < 0.01

The TMA analyses described above demonstrate that MIIP is inactivated in EC which is related to lymph node metastasis of EC. This supports our hypothesis that the *MIIP* gene functions as a migration inhibitor in EC cells, similar to what has been observed in glioma cells [10, 20]. Western blot analysis of five widely used EC cell lines showed that MIIP protein expression was relatively high in HEC1A cells but very low in AN3CA and HEC1B cells (Additional file 1: Figure S1). We examined the effects of increasing or decreasing MIIP expression on cell migration and invasion using transwell chamber assays. An adenovirus-based expression system (Ad-*MIIP*) was used to uniformly elevate MIIP expression in AN3CA and HEC1B cells, and two small-interfering RNAs (siRNA) knockdown was used to inhibit MIIP expression in HEC1A cells (Fig. 2a).

The transwell cell migration assay was done within 24 h of seeding to avoid interference of cell growth in interpretation of cell migration results. Results show that the number of migrated *MIIP*-knockdown HEC1A cells was significantly greater than that of cells transfected with the control vector (71.17 ± 3.82 and 68.86 ± 5.23 vs. 30.12 ± 1.94, *P* < 0.01). The number of migrated cells was significantly decreased in MIIP-overexpressing cells relative to negative controls for both HEC1B (21.83 ± 2.04 vs. 89.83 ± 5.56, *P* < 0.01) and AN3CA (26.67 ± 1.86 vs. 69.83 ± 2.93, *P* < 0.01) cells (Fig. 2b, c).

We also used a transwell assay to investigate whether MIIP has the same effect on invasion of HEC1A and HEC1B cells. As in the migration assay, knockdown of *MIIP* in HEC1A cells significantly increased cell invasion compared to the control cells transfected with negative control siRNA (101.34 ± 1.86 and 96.33 ± 2.64 vs 25.83 ± 1.47, Fig. 2d). While overexpression of MIIP inhibited HEC1B cell invasion significantly compared to cells transfected with negative control vector (18.33 ± 2.33 vs. 43.17 ± 4.07, Fig. 2e).

### MIIP reduces formation of lamellipodia, affects Rac1 location and inhibits Rac1 activity

**Fig. 3:**
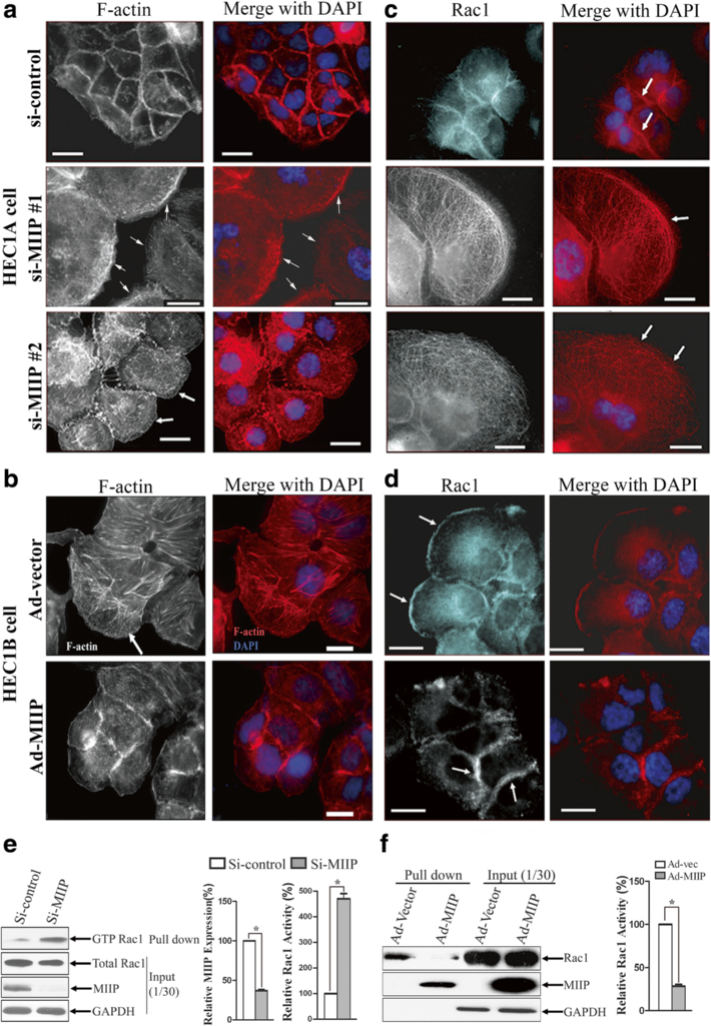
MIIP reduces formation of lamellipodia, affects Rac1 location, and inhibits Rac1 activity. **a** HEC1A cells were transfected with si-control or si-MIIP(#1and #2) for 72 h and then stained with phalloidin. *Arrow*: lamellipodia. **b** HEC1B cells were infected with Ad-*MIIP* or control adenoviral vector (Ad-Ev) at 10 multiplicity of infection (MOI) for 48 h and then stained with phalloidin (*red*). *Arrow*: lamellipodia. **c** HEC1A cells were transfected with si-control or si-MIIP (#1and #2) for 72 h and then stained with Rac1. *Arrow in the upper panel*: Rac1 location. *Arrow in the middle and lower panels*: Lamellipodia and Rac1 location. **d** HEC1B cells were infected with Ad-*MIIP* or Ad-Ev at 10 MOI for 48 h and then stained for Rac1(*red*). *Arrow in the upper panel*: Lamellipodia and Rac1 location. *Arrow in the lower panels*: Rac1 location. All images were taken by ZEISS HEO 100 microscope at a magnification of ×630. DNA was stained by DAPI (*blue*). Scale bar 10 μm. **e** Rac1 activity assay was based on the level of Rac1-GTP pulled down by the PAK1 PBD. HEC1A cells were transfected with *MIIP* siRNA or scramble control siRNA. GTP-Rac1 immunoprecipitated by GST-PAK1 PBD was detected by western blotting with an anti-Rac1-specific antibody. The *left bar chart* shows relative levels of MIIP, and the *right bar chart* shows relative levels of Rac1-GTP. Rac1 activity is presented as percentage of GTP-Rac1 compared to total Rac1 (as 100 %). Data are shown as means ± SD from three independent experiments; *P < 0.01 (*t* test). **f** Rac1 activity assay was performed on HEC1B cells overexpressing MIIP. HEC1B cells were infected with Ad-*MIIP* or control adenoviral vector (Ad-Ev). GTP-Rac1 immunoprecipitated by GST PAK1 PBD domain was detected by western blotting with Rac1 antibody. MIIP was also pulled down by PAK1. The *bar chart* shows relative Rac1 activity. Data are shown as means ± SD from three independent experiments; *P < 0.01 (*t* test)

Lamellipodia, broad sheet-like protrusions containing a network of branching actin filaments, are found at the front of migrating cells and drive cell migration. To gain insight into the molecular basis for the effect of MIIP on cell migration, we evaluated structural changes in the actin cytoskeleton by staining F-actin in HEC1A cells before and after *MIIP* knockdown. *MIIP*-knockdown cells had extensive lamellipodia at their leading edges compared to the controls (Fig. 3a). In *MIIP*-overexpressing HEC1B cells, lamellipodia formation was consistently reduced significantly compared to that in control cells (Fig. 3b).

Because Rac1 is a well-known molecule in regulation of lamellipodia structure, we performed immunofluorescence staining of Rac1. We observed large lamellipodia with Rac1 concentrated on the leading edge in *MIIP*-knockdown HEC1A cells, whereas lamellipodia were rarely observed in control HEC1A cells and Rac1 was concentrated mainly at the cell-cell borders (Fig. 3c). Consistent with this finding was the observation of large lamellipodia with Rac1 concentrated on the leading edge in the control HEC1B cells, but lamellipodia were observed only rarely in *MIIP*-overexpressing HEC1B cells and Rac1 was concentrated mainly at the cell-cell borders (Fig. 3d).

We next evaluated whether MIIP modulated Rac1 activity in the EC cells. We performed a Rac activity assay to evaluate the levels of activated Rac1 by using pull-down Rac1-GTP with the PAK1 p21-binding domain (PBD). The level of GTP-bound activated Rac1 was significantly higher in *MIIP*-knockdown HEC1A cells than in control cells (Fig. 3e). Moreover, the level of GTP-bound activated Rac1 in MIIP-overexpressing HEC1B cells was consistently lower than that in the controls (Fig. 3f).

### MIIP blocks Rac1 signaling by directly binding to its downstream effector PAK1

**Fig. 4:**
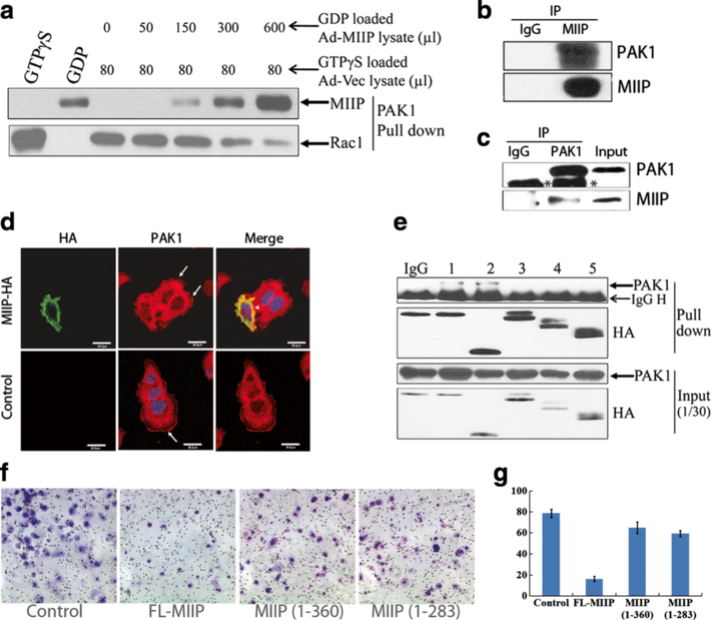
MIIP blocking of cell migration through competing with Rac1-GTP to bind the PAK1-binding domain. **a** Competition for PAK1 PBD binding between MIIP and Rac1. HEC1B cell lysate with MIIP loaded with GDP (different amounts of MIIP) and control HEC1B lysate loaded with GTPγS (same amounts of Rac1/GTP) were incubated with GST-PAK1 PBD-coupled agarose beads, and the pulled down complex was detected by indicated antibodies. The original cell lysate loaded with GTPγS or GDP was used as positive or negative control, respectively. **b** In an immunoprecipitation assay using anti-MIIP antibody, PAK1 was pulled down by MIIP. **c** In a reverse immunoprecipitation assay using anti-PAK1 antibody, MIIP was pulled down by PAK1. *Asterisk* indicates IgG heavy chain. **d** Co-localization of MIIP and PAK1 in HEC1B cells. HEC1B cells were transfected with HA-tagged *MIIP* for 48 h and then stained with HA (*green*) and PAK1 (*red*). All images were taken by ZEISS HEO 100 microscope at a magnification of ×630. Scale bar 10 μm. *Arrow*: lamellipodia. *Arrow head*: MIIP and PAK1 co-localization. **e** Immunoprecipitation assays used rabbit anti-HA in HEC1B cells transfected with wild-type *MIIP* or one of the four truncated *MIIP* constructs. Input: immunoblot of a steady level of MIIP, Rac1, or PAK1 in HEC1B cell lysates (30 % of the amount of the same cell lysate sample used for immunoprecipitation). *IgG H* indicates IgG heavy chain. C, 1, 2, 3, 4, and 5 indicate control, wild-type MIIP, MIIP (155-388), MIIP (1-360), MIIP (1-313), and MIIP (1-283), respectively. **f**, **g** The effects of different truncated MIIPs on cell migration. **f** Representative photographs of different truncated MIIPs on cell migration by transwell migration assay (magnification ×200). **g** Data are expressed as means ± SD of cells per 10 high-power fields (magnification ×200) from three separate experiments

To understand whether MIIP’s modulation of Rac1-GTP level is mediated by blocking its binding to the PAK1 PBD, we loaded GTPγS (which would form activated GTP-Rac1) to lysates of untreated HEC1B cells and GDP (which would convert Rac1-GTP to Rac1-GDP) to lysates of MIIP-overexpressing HEC1B cells and, using a Rac1 activation assay kit, carried out a competition binding assay in which MIIP concentration was increased gradually while GTP-Rac1 protein input remained constant. The original cell lysate with GTPγS or GDP loading was used as a positive or negative control, respectively. The results showed that increasing MIIP level gradually blocked GTP-Rac1 binding to the PAK1 PBD, suggesting that MIIP competed with Rac1 for binding to the PAK1 PBD (Fig. 4a).

To further confirm that MIIP binds to PAK1 in EC cells, we performed co-immunoprecipitation analysis (co-IP) by using MIIP antibody with total proteins extracted from MIIP-overexpressing HEC1B cells. The results show that PAK1 protein was pulled down by MIIP (Fig. 4b). We also performed reverse co-IP by using PAK1 antibody with total proteins extracted from HEC1A cells. The results show that endogenous MIIP protein was pulled down by PAK1 (Fig. 4c). Our co-immunofluorescence assay data showed that MIIP and PAK1 are co-localized in HEC1B cells (Fig. 4d). To further characterize the binding domains of MIIP that interact with PAK1, hemagglutinin (HA)-tagged expression vector with wild-type MIIP, or with one of four truncated MIIPs, was transfected into HEC1B cells for 24 h followed by co-IP to examine the binding with PAK1. An HA antibody was used to immunoprecipitate PAK1 protein, and an anti-HA antibody was used to detect MIIP protein in the immunoprecipitated complexes. As shown in Fig. 4e, the wild-type MIIP could bind to PAK1. MIIP with an N-terminal deletion (153-388) exhibited strong binding to PAK1, whereas MIIP with a C-terminal deletion (1-360, 1-313, or 1-283) showed no detectable binding to PAK1. Thus, the C-terminal region of MIIP (360 ASPMQMLPPTPTWSVPQVPR PHVPRQKP 388), containing a polyproline domain, is required for binding to PAK1. We further evaluated the effect of the PAK1-binding domain of MIIP on cell migration, and the data show that loss of PAK1-binding domain reduced inhibition of HEC1B cell migration (Fig. 4f and g).

## Discussion

**Fig. 5:**
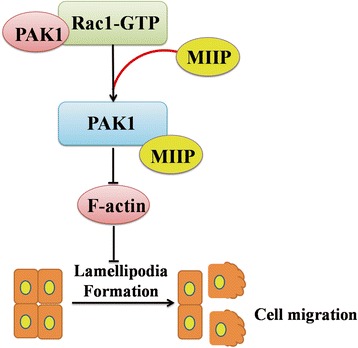
MIIP competing with Rac1-GTP to bind the PAK1-binding domain. The results of our studies provide evidence that MIIP inhibited EC migration through reducing the formation of lamellipodia. MIIP may block Rac1 signaling pathway through competing binding to Rac1’s downstream effector PAK1, which resulted in decreasing formation of lamellipodia and then inhibiting EC cell migration

In this study, we investigated the biological function and clinical significance of *MIIP* in EC progression through a TMA MIIP expression. The results of our studies provide evidence that MIIP inhibited EC migration through resulting in cytoskeleton reorganization with markedly decreased formation of lamellipodia. MIIP may block Rac1 signaling pathway through competing binding to Rac1’s downstream effector PAK1, which resulted in decreasing formation of lamellipodia and then inhibiting EC cell migration (Fig. 5). Specifically, we demonstrated that a C-terminal polyproline domain of MIIP is required for PAK1 binding.

This newly recognized TSG has obvious clinical significance in EC patients. We found that *MIIP* expression is deceased in EC tissues, especially in patients with deep myometrial invasion, advanced stage, and lymph node metastasis which is consistent with a previous study reporting decreased MIIP in advanced gliomas [11]. MIIP inactivation was also reported in breast, esophageal, and lung cancer [19, 21, 22]. Thus, MIIP was considered to play a major role in inhibiting epithelial tumor metastasis, which includes EC, and the inactivation of the *MIIP* gene in EC prompted us to further investigate its tumor suppressor functions.

Highly augmented cell motility is a fundamental aspect of increased metastasis in cancer, a process well recognized as the primary killer of cancer patients. Our previous studies showed that MIIP binds and inhibits HDAC6, which leads to acetylation of alpha tubulin and stabilization of microtubules, slowing cell migration [11]. When we modulated MIIP levels in EC cells, however, we were surprised that we did not detect significant changes in tubulin acetylation even though the effect on cell migration was evident. Moreover, we observed remarkable changes in lamellipodia in these cells when MIIP expression was modulated. Coordinations between the actin cytoskeleton and microtubules are crucial for cell polarization, shape changes, and migration. During cell migration, the actin cytoskeleton is thought to provide the driving force. At the leading edge of the cell, actin is organized in a dense meshwork which forms lamellipodia and promotes forward movement. In most cell types, migration is altered by interactions between the microtubule and actin cytoskeleton. However, migration of fish keratocytes is unaffected by microtubule disassembly, and neutrophil motility is even increased in the absence of microtubules [23]. Our previous work showed that MIIP could inhibit cancer cell migration in several types of cancer cells. Our results revealed similar function of MIIP in EC cells and that actin cytoskeleton reorganization and decreased formation of lamellipodia were associated with over-abundant MIIP, which likely lead to inhibition of metastasis.

Various regulators of the actin cytoskeleton are involved in the invasive and metastatic phenotypes. Rho GTPases are master regulators of actin structures and dynamics [12, 24]. Our previous study indicated that Rho GTPase family members were downregulated in MIIP-expressing cells [10]. Rac1, one of the best known small GTPases, integrates the upstream signals from extracellular stimuli, including integrins, hormones, growth factors, and cytokines [25], relays signals to downstream kinase PAK [26], and then promotes lamellipodia formation and cell migration. This led us to investigate the effect of MIIP on Rac1 signaling pathway. The increased motility is an energy-costing process that is clearly in high gear in metastatic EC [27]. Understanding how this engine is fired up and what can be done to block this key step is of fundamental importance for understanding cancer progression and for developing effective therapeutic strategies. In this study, we demonstrate that the recently identified MIIP is a key player, controlling the Rac1 signaling pathway at the critical junction where GTP-Rac1 interacts with its downstream effector proteins PAK1. PAK1 plays an important role in remodeling of the cytoskeleton and promoting increased cell motility [28]. In the present study, we observed that the PAK1-binding fraction of Rac1, Rac1-GTP, was reduced when MIIP expression was elevated. Specifically, we provided evidence that MIIP directly binds to PAK1, and this binding attenuates interaction of its mediated proteins with its upstream GTPase Rac1 and blocks Rac1/PAK1 signaling, inhibiting EC cell migration.

Results from this and our previous studies have shown that MIIP is a key inhibitor of cell migration and regulates multiple related steps. We showed previously that MIIP binds to IGFBP2 and inhibits IGFBP2-mediated cell migration [10], which requires the interaction of IGFBP2 with integrin α5, which activates Rac1 [29, 30]. This suggests that MIIP negatively regulates the integrin-cytoskeleton pathway at least two junctions (one upstream and one downstream). We also showed that MIIP blocks HDAC6-mediated cell migration [11]. Interestingly, the F-actin-binding protein cortactin was recently shown to be a substrate for HDAC6 [31]. Further, it has been reported that microtubule growth and shortening can activate Rac1 and RhoA signaling to control actin dynamics [32]. Therefore, inhibition of HDAC6 can lead to attenuation of the actin cytoskeleton-mediated cell migration signaling pathway, either directly or through the microtubule-mediated cytoskeleton pathway [33]. Thus, it is conceivable that a main target for MIIP regulation is the actin cytoskeleton system, at least in EC cells.

Our findings suggest that restoration of MIIP may have critical importance for effective treatment of ECs. We found that MIIP expression is decreased in ECs, especially in deep myometrial invasion, or advanced stage cases and those with lymph node metastasis. High-grade EC has a greater tendency to be myometrial invasion and associated with lymphatic metastasis than low-grade tumors and is associated with poor responsiveness to radiation, chemotherapy, and hormone therapy [34–36]. Along this line, the fact that we found the C-terminal polyproline-rich region of 28 amino acids to be responsible for binding to PAK1 may provide a starting point for evaluating peptide-based inhibitors that block the Rac1 signaling pathway in endometrial and possibly other types of cancers.

Although the present study reported a novel mechanism which *MIIP* inhibits EC cell motility through blocking of the Rac1 signal transduction pathway by directly binding to its downstream effector PAK1, there are still some questions. First, except for Rac1, RhoA and CDC42 are required for actin cytoskeleton remodeling too. The GTPase cross-talks in cell migration is complex, so it will be interesting to figure out when and where *MIIP* plays its role in Rho GTPases’ signaling pathway. Second, it will be important to determine preciously how MIIP coordinates the crosslink between actin and microtubules cytoskeleton organization to promote cell migration. Finally, our experiments showed that MIIP could reduce MMP9 (Additional file 2: Figure S2), which is consistent with the recent report of Rac1/Pak1/p38/MMP axis in ovarian cancer oncogenesis [37]. All of these deserved further study in the future.

## Conclusions

In summary, the identification of MIIP as an inhibitor of Rac1 signaling by competitive binding to its downstream effector protein PAK1 has expanded our understanding of these pathways and shed light on how the cell migration pathways can be activated in cancer. This investigation also provides a foundation for further research into cancer therapeutics exploring the MIIP protein and its mechanism of action.

## Methods

(Selected materials and methods are described here. The detailed procedures for all related experiments are can be found in Additional file 3).

### Antibodies, plasmids, and reagents

We used a rabbit anti-human MIIP polyclonal antibody raised against *MIIP* epitope (46 NSETPSTPETSSTSL 60) as described elsewhere [10]. The polyclonal antibody against GAPDH antibody and the anti-αPAK antibody-coupled agarose beads were purchased from Santa Cruz Biotechnology (Santa Cruz, CA, USA). Monoclonal anti-HA-agarose beads were obtained from Sigma-Aldrich (St. Louis, MO, USA). Antibodies for Rac1 and PAK1 were purchased from Cell Signaling Technology (Boston, MA, USA). The Alexa Fluor 488 goat anti-mouse IgG (H + L) (cat# A11029) and the Alexa Fluor 594 goat anti-rabbit IgG (H + L) (cat# A11037) were obtained from Invitrogen (Carlsbad, CA, USA). Transfection was performed with Lipofectamine 2000 (Invitrogen) by following the manufacturer’s instructions. SiRNA for MIIP (ID: #1-123298 and #2-127111) and for the nontargeting control were obtained from Ambion (Austin, TX, USA).

### Patient samples

Endometrial tissue samples from Tianjin Medical University General Hospital (Tianjin, China) were collected between 2003 and 2014 from 205 patients with EC, 63 patients with atypical hyperplasia, and 116 patients with normal endometrium. All of the tissues were collected from hysterectomy specimens. The patients without EC in our study were admitted to our hospital for uterine prolapse, cystocele, or urethrocele. The histological type and grade of the tumors were determined based on a modified WHO classification system, and EC staging was performed based on a modified 2009 FIGO staging system. The protocol of the study was reviewed and approved by the Ethics Committee of Tianjin Medical University General Hospital.

### Immunohistochemistry

Immunohistochemical analysis was performed for MIIP on TMA using a previously described method [38].

### Cell culture

The EC cell lines AN3CA, ECC-1, HEC1A, and HEC1B were obtained from American Type Culture Collection (Manassas, VA, USA). The Ishikawa cell line was kindly provided by Dr Kim K Leslie (The University of New Mexico Health Sciences Center, Albuquerque, NM, USA). HEC1A was maintained in McCoy’s 5A medium, while the other four cell lines were maintained in Eagle’s minimum essential medium, both supplemented with 10 % (*v*/*v*) fetal bovine serum (FBS), 100 U/ml penicillin, and 100 μg/ml streptomycin. All cells were incubated at 37 °C in a humidified atmosphere of 5 % CO_2_.

### Western blotting analysis

Cell extracts containing 40–80 μg of protein were resolved by 10 % sodium dodecyl sulfate polyacrylamide gel electrophoresis (SDS-PAGE), transferred to Hybond ECL nitrocellulose membranes (Amersham Pharmacia Biotech, Chicago, IL, USA), blocked in 5 % nonfat milk in 1× Tris-buffered saline (pH 8.0) containing 0.05 % Tween-20, and probed with the primary antibodies at concentrations of 1:1000. The secondary antibodies were used at concentrations of 1:10,000 to 1:50,000. The proteins were visualized using the SuperSignal West Pico Chemiluminescent Substrate (Pierce Chemical, Rockford, IL, USA).

### Transwell cell migration/invasion assays

Assays were performed on transwell culture slides with uncoated porous filters (8.0-μm pore size) (Corning Life Sciences, Acton, MA, USA) and filters (8.0-μm pore size) precoated with Matrigel (Becton Dickinson Labware, Franklin Lakes, NJ, USA) to examine cell migration and invasion as described previously [39]. Briefly, the main difference between migration and invasion examination methods lied in whether the filters were coated with Matrigel. In order for cell invasion to occur, the cells have to secrete enzymes to digest the gelatinous protein mixture in the Matrigel before moving to the other side of the chamber.

### Immunoprecipitation

The experiments were performed using 100 μl of Dynabeads M-280 sheep anti-rabbit IgG or sheep anti-mouse IgG (Invitrogen, #112-03D or #112-01D), and the manufacturer’s instructions were followed.

### Immunofluorescence staining

Assays were performed as described elsewhere [40].

### Rac activity assay

Rac activity levels were measured by using the GST PAK1/PBD pull-down assay (Cell Biolabs, Inc., San Diego, CA, USA) according to the manufacturer’s instructions.

### Statistical analysis

All data are shown as the mean ± SD. Differences between *MIIP* loss/gain and negative controls were analyzed by using Student’s *t* test. Clinical data were analyzed by using chi-square or one-way ANOVA with least significant difference or Tamhane’s T2 post hoc test to assess differences between experimental groups. The statistical software used was SPSS, version 16.0 (SPSS Inc, Chicago, IL, USA). *P* values of less than 0.05 were considered statistically significant.

## Additional files

Additional file 1: Figure S1. The expression of MIIP in five widely used EC cell lines. (DOC 38 kb)
Additional file 2: Figure S2. MIIP reduces MMP9 level. (DOC 137 kb)
Additional file 3: Supplemental methods. Supplemental information of methods. (DOC 262 kb)

## Abbreviations

AHE: Atypical hyperplasia endometrium (AHE)
CDC42: Cell division cycle 42
EC: Endometrial carcinoma
GDP: Guanosine diphosphate
GTP: Guanosine triphosphate
HA: Hemagglutinin
HDAC6: Histone deacetylase 6
IGFBP2: Insulin-like growth factor-binding protein 2
IP: Immunoprecipitation
MIIP: Migration and invasion inhibitory protein
MSI: Microsatellite instability
NE: Normal endometrium
PAK1: p21-Activating kinase 1
PBD: p21-Binding domain
PTEN: Phosphatase and tensin homolog
Rac1: Ras-related C3 botulinum toxin substrate 1
RhoA: Ras homolog family member A
siRNA: Small-interfering RNAs
TCGA: The Cancer Genome Atlas
TP53: Tumor protein p53
