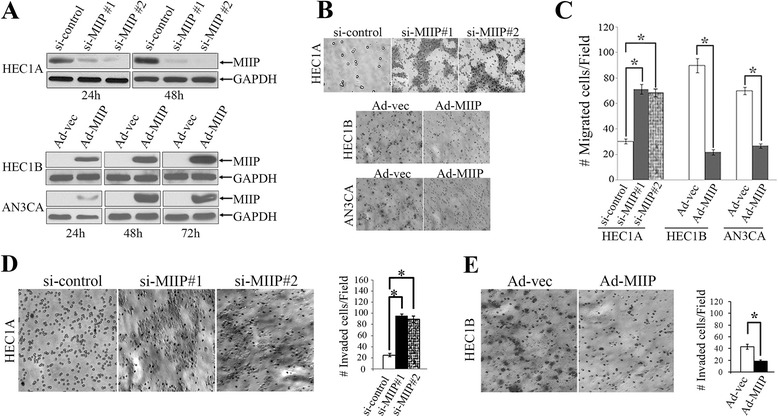# Erratum to: MIIP remodels Rac1-mediated cytoskeleton structure in suppression of endometrial cancer metastasis

**DOI:** 10.1186/s13045-017-0525-9

**Published:** 2017-10-06

**Authors:** Yingmei Wang, Limei Hu, Ping Ji, Fei Teng, Wenyan Tian, Yuexin Liu, David Cogdell, Jinsong Liu, Anil K. Sood, Russell Broaddus, Fengxia Xue, Wei Zhang

**Affiliations:** 1 Department of Gynecology and Obstetrics, Tianjin Medical University General Hospital, Tianjin, China; 2 Department of Pathology, The University of Texas MD Anderson Cancer Center, Houston, TX USA; 3 Department of Systems Biology, The University of Texas MD Anderson Cancer Center, Houston, TX USA; 4 Department of Bioinformatics, The University of Texas MD Anderson Cancer Center, Houston, TX USA; 5 Department of Gynecologic Oncology and Reproductive Medicine, The University of Texas MD Anderson Cancer Center, Houston, TX USA; 6 Center for RNAi and Non-Coding RNA, The University of Texas MD Anderson Cancer Center, Houston, TX USA; 7 Department of Cancer Biology, Comprehensive Cancer Center of Wake Forest Baptist Medical Center, Winston-Salem, NC 27157 USA; 8 Present Address: Department of Biochemistry and Molecular Biology, The University of Texas Medical Branch, Galveston, TX USA

## Erratum

The original article [1] contains an error whereby Fig. 2D mistakenly displays a duplicate image for the panels, ‘si-MIIP#1’ and ‘si-MIIP#2’.

The correct version of Fig. 2D and its constituent panels can instead be viewed below.