# Supplementary material for: MIIP remodels Rac1-mediated cytoskeleton structure in suppression of endometrial cancer metastasis
Source: J Hematol Oncol. 2016 Oct 19;9:112. doi: 10.1186/s13045-016-0342-6 (PMC5069779; doi:10.1186/s13045-016-0342-6)
Supplement: Additional file 1: Figure S1. — The expression of MIIP in five widely used EC cell lines. (DOC 38 kb) [file 13045_2016_342_MOESM1_ESM.doc]

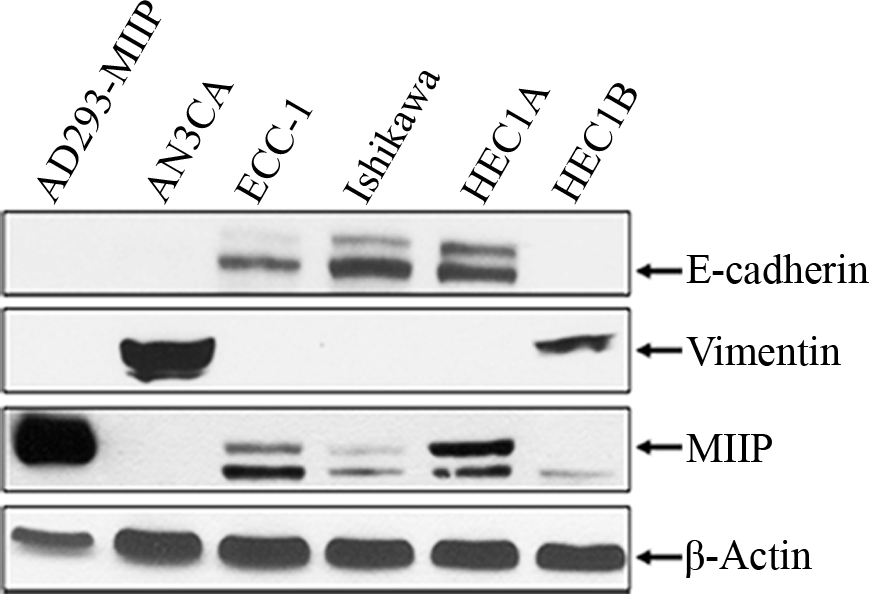


**Additional file 1: Figure S1.** The expression of MIIP in five widely used EC cell lines, including AN3CA, ECC-1, Ishikawa, HEC1A, and HEC1B. MIIP protein expression levels were relatively high in HEC1A cells but low in AN3CA and HEC1B cells, which exhibit mesenchymal cell characteristics: low expression of epithelial cell marker E-cadherin and higher expression of mesenchymal cell marker vimentin AD293-MIIP were positive controls for MIIP.
