# Supplementary material for: MIIP remodels Rac1-mediated cytoskeleton structure in suppression of endometrial cancer metastasis
Source: J Hematol Oncol. 2016 Oct 19;9:112. doi: 10.1186/s13045-016-0342-6 (PMC5069779; doi:10.1186/s13045-016-0342-6)
Supplement: Additional file 2: Figure S2. — MIIP reduces MMP9 level. (DOC 137 kb) [file 13045_2016_342_MOESM2_ESM.doc]

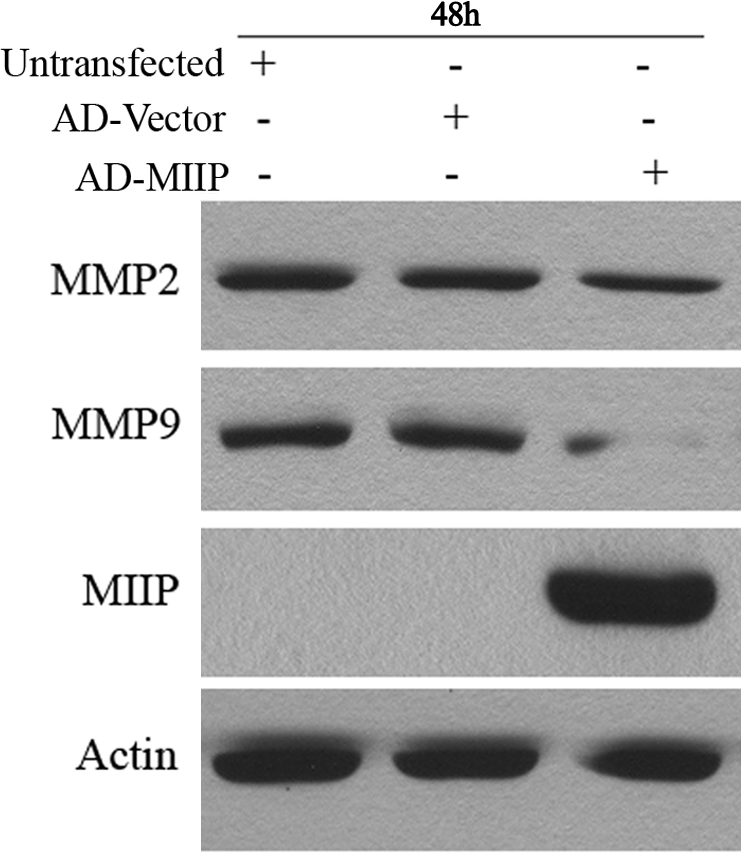


**Additional file 2: Figure S2.** MIIP expression was forced in HEC1B cells by infection with an adenovirus containing *MIIP* (Ad-*MIIP*) or control adenovirus (Ad-Vector) at 48h. MIIP overexpression could reduce MMP9 expression levels significantly but not MMP2.
