# Supplementary material for: MIIP remodels Rac1-mediated cytoskeleton structure in suppression of endometrial cancer metastasis
Source: J Hematol Oncol. 2016 Oct 19;9:112. doi: 10.1186/s13045-016-0342-6 (PMC5069779; doi:10.1186/s13045-016-0342-6)
Supplement: Additional file 3: — Supplemental methods. Supplemental information of methods. (DOC 262 kb) [file 13045_2016_342_MOESM3_ESM.doc]

**Supplemental Methods**

*Immunohistochemical analysis*

In brief, TMA sections were incubated in rabbit polyclonal anti-MIIP antibody (1:200; Sigma, St. Louis, MO, USA) overnight at 4°C in humidified chambers. The following day, the sections were washed three times in phosphate-buffered saline (PBS) and incubated in peroxidase-conjugated goat anti-rabbit antibody IgG antibody, which had been obtained from a streptavidin-peroxidase-biotin reagent kit (Zhongshan Biotechnology Company, Beijing, China), for 30 minutes at 37°C. After being washed in PBS, the tissue sections were stained with diaminobenzidine, counterstained with hematoxylin, and examined under a light microscope. As negative controls, tissue sections were processed as described above, except that they were incubated overnight at 4°C in blocking solution with PBS. MIIP staining was scored on the basis of the percentage and intensity of positively stained cells. The five scoring categories for the positive staining percentage were as follows: 0, no positive cells; 1, 25% or fewer positive cells; 2, 26% to 50% positive cells; 3, 51% to 75% positive cells; and 4, 76% or more positive cells. The four scoring categories for staining intensity were as follows: 0, no intensity; 1, weak intensity; 2, moderate intensity; and 3, strong intensity. High MIIP expression was defined as ≥ 3, and low expression was defined as ≤ 3.

*Transwell cell migration and invasion assays*

HEC1A cells were transfected with si-MIIP (#1 and #2) or si-control, while AN3CA and HEC1B cells were infected with Ad-MIIP or Ad-Ev as a negative control for 24 h and cultured in the upper chamber at a final concentration of 1×10^5^/100 μl in FBS-free medium; 500 μl or 750 μl of the medium, supplemented with 20% FBS, was added to the lower chamber. After 24 h of incubation, the remaining tumor cells on the upper surface of the filters were removed by wiping with cotton swabs, and the migrated and invaded cells on the lower surface of the filters were fixed and stained with Giemsa. The migrated and invaded cells were counted using a microscope at a magnification of ×200. Data were obtained from three individual experiments performed in triplicate.

*Immunoprecipitation*

In brief, after being washed with 1× PBS three times, 2 μg of antibody or normal rabbit or mouse IgG in 1× lysis buffer (Cell Biolabs, Inc., San Diego, CA, USA; cat #240102) containing 1× protease inhibitor cocktail (Sigma, cat #P8340) was added to the corresponding tubes, which were rotated at 4°C for 1 h, followed by the addition of 1 ml of cell lysate containing about 1 mg of protein. The binding of the protein and antibody was allowed to continue at 4°C overnight. The beads were then washed three times with 1× lysis buffer, and the immunoprecipitated proteins were visualized by western blot analysis. The original cell lysates were used for protein expression as input quantification (1/30 of the total lysate of each sample) using GAPDH as the loading control.

*Immunofluorescence staining*

In brief, cells were fixed with 4% paraformaldehyde in PBS, permeabilized with 0.1% Triton X-100 in PBS for 10 min, and blocked with 10% normal goat serum for at least 1 h at ambient temperature. For single-antibody staining, the cells were incubated with fluorescence-conjugated antibody at a concentration of 1:100 for 1 h at ambient temperature. For double staining, the cells were incubated with the first antibody at a concentration of 1:1000 for 1 h at ambient temperature or for 16-18 h at 4°C. After being washed, the cells were incubated with the fluorescence-conjugated secondary antibody at a concentration of 1:100 for 1 h at ambient temperature. After another washing, they were counterstained with DAPI for fluorescence microscopy. Phalloidin staining was performed at ambient temperature for 45 min using Texas Red-conjugated phalloidin (Molecular Probes, Invitrogen) at a concentration of 5 µg/ml. Images were captured by confocal or phase-contrast fluorescence microscopy.

*Rac activity assay*

In brief, HEC1B cells were seeded (1 × 10^6^/10-cm dish) and incubated for 24 h before being infected with Ad-MIIP or Ad-Ev (MOI = 10) for 48 h. For siRNA treatment experiments, HEC1A cells were seeded (4 × 10^6^/10-cm dish) and incubated for 24 h before being transfected with pooled *MIIP* siRNA or control siRNA and incubated for another 24 h; they were then trypsinized and seeded to a new 10-cm dish for 48 h of incubation. The cells were washed twice with ice-cold PBS and subjected to lysis with 1 × lysis buffer (Cell Biolabs, Inc., cat #240102) containing 1× protease inhibitor cocktail (Sigma, cat #P8340). One-milliliter volumes were taken from each cell lysate sample, 40 μl of beads with GST-PAK1-PBD were added to each tube, and the tube was rotated at 4°C for 1 h. The beads were then washed three times with lysis buffer, and the pulled down GTP-Rac1 was detected by specific Rac1 antibody. The GTPγS- and GDP-loaded samples were used as positive and negative controls, respectively.

For the competition binding assays, we used the same beads used for the active Rac pull-down assay described above. HEC1B cells were either infected with Ad-*MIIP* (MOI = 10) for 48 h or untreated. The lysate from untreated cells was loaded with GTPγS; to avoid the effect of the active form of Rac on MIIP-overexpressing cells, the lysate from MIIP-overexpressing cells was loaded with GDP. GDP-loaded MIIP-overexpressing cell lysate (0, 50, 150, 300, or 600 μl) was added into each tube containing 80 μl of the GTPγS-loaded cell lysate. The pull-down assays were performed as described in the previous paragraph (Rac activity assay).
